# Searching for a Solution: A Case Report on Multifocal Ectopic Purkinje-Related Premature Contractions Syndrome

**DOI:** 10.3390/medicina61030393

**Published:** 2025-02-24

**Authors:** Monika Keževičiūtė, Neringa Bileišienė, Violeta Mikštienė, Germanas Marinskis, Jūratė Barysienė

**Affiliations:** 1 Clinic of Cardiac and Vascular Diseases, Institute of Clinical Medicine, Faculty of Medicine, Vilnius University, Santariskiu str. 2, LT-08661 Vilnius, Lithuania; neringa.bileisiene@santa.lt (N.B.); germanas.marinskis@santa.lt (G.M.); jurate.barysiene@santa.lt (J.B.); 2 Department of Human and Medical Genetics, Institute of Biomedical Sciences, Faculty of Medicine, Vilnius University, Santariskiu g. 2, LT-08661 Vilnius, Lithuania; violeta.mikstiene@santa.lt

**Keywords:** multifocal ectopic Purkinje-related premature contractions, SCN5A, mutation, ventricular tachycardia, dilated cardiomyopathy, case report

## Abstract

Multifocal ectopic Purkinje-related premature contractions (MEPPC) syndrome is a recently recognized rare form of arrhythmia involving the entire His–Purkinje system and often coinciding with dilated cardiomyopathy (DCM). Certain variants in the SCN5A gene may be linked to MEPPC syndrome. We present a case of a 32-year-old Caucasian female who exhibited a high burden of premature ventricular contractions (PVCs) and non-sustained episodes of ventricular tachycardia (NSVT) with an alternating QRS pattern, and who was resistant to traditional medical therapy and radiofrequency catheter ablation (RFCA), necessitating implantation of a cardioverter-defibrillator (ICD). A positive family history (father’s death at the age of 40 years) and the rapid deterioration of left ventricular function parameters echocardiographically during recurrent arrhythmic episodes raised concern about a potentially complex disease scenario. Genetic testing revealed a heterozygous variant of the SCN5A gene, *c.2440C>T*, p.(Arg814Trp), confirming the diagnosis of MEPPC syndrome. Treatment with a combination of class I antiarrhythmic drugs, flecainide and mexiletine, concomitant with beta blockers, led to symptomatic improvement, a reduction of PVCs (from 66 491 (44%) to 858 (1%)), and the restoration of left ventricular function (LV EF from 44% to 53%). A lack of defined diagnostic criteria hampers timely diagnosis, leading to ineffective interventions and delayed initiation of treatment with antiarrhythmic drugs. MEPPC patients remain at significant risk for severe heart failure and sudden cardiac death. Our clinical case report underscores the importance of accurate and timely diagnosis, which allows effective treatment with a combination of antiarrhythmic drugs and mitigates the risk associated with MEPPC syndrome.

## 1. Introduction

MEPPC syndrome is a very rare complex electrical disorder characterized by a high burden of frequent multifocal ectopic activity originating from the entire His–Purkinje system and is often accompanied by dilated cardiomyopathy [1]. Typical syndrome presentation is polymorphic premature ventricular contractions with relatively narrow QRS interval, accompanied by the patient’s complaints of palpitations, physical exercise intolerance, or syncope. In some cases, the first and the only presentation may be a sudden cardiac death [2]. Clinical symptoms frequently manifest at a young age. The incidence of the disease is not known due to its rare nature and potentially underdiagnosed status. Genetic research has identified specific gene mutations in the *SCN5A* gene, demonstrating an autosomal dominant inheritance pattern with almost complete penetrance [3]. However, the diagnosis of MEPPC is challenging due to the lack of clearly defined diagnostic criteria. Suspicion should arise when a high burden of polymorphic PVCs, typically with narrow QRS complexes or branch block patterns, is documented. These PVCs are accompanied by structural changes in the heart, in most cases ventricular dilation, as well as impaired left ventricular systolic function [4]. While catheter ablation, the standard treatment for PVC-induced cardiomyopathy, has limited efficacy in eradicating MEPPC-associated PVCs due to their diffuse origin within the Purkinje system, class 1 antiarrhythmic drugs (AADs), particularly flecainide and quinidine, have demonstrated advantageous effects in suppressing PVCs and potentially reversing DCM [5,6]. Despite this fact, MEPPC patients remain at significant risk for severe heart failure and sudden cardiac death [1]. Moreover, the clinical course of the disease may be aggravated by overlap syndromes characterized by mixed phenotypes, as mutations in voltage-gated sodium channels, particularly the NaV1.5 protein encoded by the SCN5A gene, have been associated with a number of conditions such as sick sinus syndrome, atrial standstill, atrial fibrillation, progressive and non-progressive cardiac conduction disease, Brugada syndrome, sudden infant cardiac death (SCD), long QT syndrome type 3 (LQTS3), and torsade de pointes ventricular tachycardia [7,8].

With this paper, we aim to emphasize the utility of cardiogenetics knowledge in establishing connections between certain mutations and distinct phenotypes. By doing so, we can achieve a prompt diagnosis and implement specific treatment approaches. This proactive approach can aid in mitigating the risks associated with MEPPC syndrome.

## 2. Materials and Methods

The patient gave her written informed consent for this case report, which has been approved by The Ethics Committee of Vilnius University Hospital Santaros Clinics (VUH SK).

The following personal data of the subject were processed: previous medical and family history, standard 12-lead electrocardiogram (ECG), transthoracic echocardiography (TTE), cardiac magnetic resonance, stress test, 24 h Holter monitoring, and electrophysiological procedures.

Our patient underwent genetic testing. Clinical evaluation consisted of previous medical and family history, standard 12-lead ECGs, TTE, exercise testing, and 24 h Holter monitoring.

This clinical case includes a comprehensive review of the literature relevant to the topic. The MED-LINE database was used for a search of highly regarded articles from 2005 to 2025. Keywords, including “MEPPC syndrome”; “SCN5A gene”; and “premature ventricular contraction” were used. Papers from the past five years were prioritized. The bibliography was composed using Mendeley Reference Management software, version 2.125.2.

## 3. Case Report

**Figure 1:**
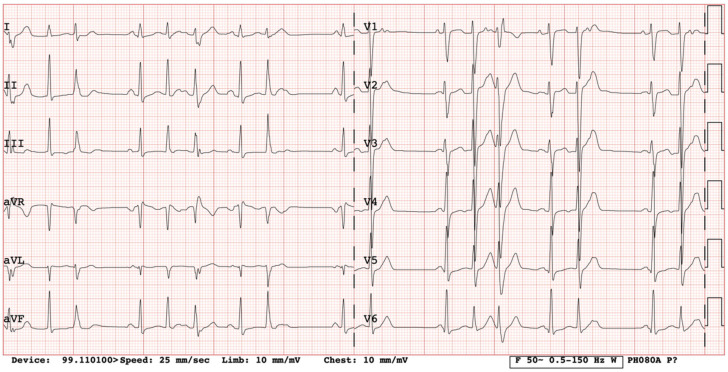
Twelve-lead ECG on admission (frequent polymorphic PVCs).

**Figure 2:**
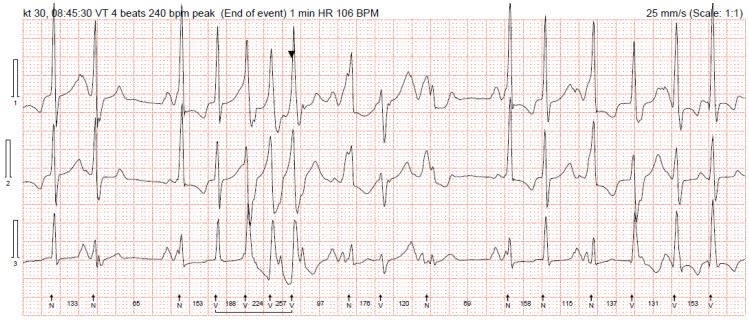
Holter 24 h monitoring (episode of NSVT).

We present a clinical case of a 32-year-old Caucasian woman. Since the age of 17 years, she had complaints of palpitations and physical activity intolerance. The patient underwent a comprehensive clinical examination at the University Hospital. Laboratory and imaging tests excluded structural heart disease, electrolyte imbalance, and other possible causes of arrythmias. In our case, most of the PVCs originated from the right ventricular outflow tract (monomorphic); thus, the first catheter ablation of PVCs was performed at the age of 17. For the recurrence of arrhythmia, a monotherapy of metoprolol and a later monotherapy of propafenone were assigned but proved ineffective. After two years of ineffective medical therapy, the patient underwent a second electrophysiological testing but no target zones of arrhythmia circuits were identified. She was seen again by a cardiologist at the age of 20 years. She complained of dizziness and irregular heartbeats. Frequent PVCs in her ECG were registered (Figure 1). A bicycle ergometer stress test was consistent with ventricular bigeminy, several couplets, and NSVT episodes of 3–4 ventricular complexes. Holter 24 h monitoring registered 47,000 (40%) premature supraventricular contractions and 14,137 (13%) PVCs, where 226 of them were couplets with a morphology consistent with right ventricular origin. Cardiac magnetic resonance was performed. The evaluation of images was difficult because of arrhythmic artefacts. The imprecise left ventricle ejection fraction (LV EF) was around 34%, LVdd was 55 mm, and no significant late gadolinium enhancement was evident. Brain natriuretic peptide was 170.4 ng/L and she was clinically assigned to New York Heart Association (NYHA) class II. A partial reduction of PVCs was observed with beta blockers at that time, and a mineralocorticoid receptor antagonist was added to her treatment (spironolactone at a dose of 25 mg per day). It was decided to implant a dual-chamber implantable cardioverter-defibrillator (ICD) and, at discharge, antiarrhythmic therapy consisted of 200 mg of amiodarone and 100 mg of metoprolol per day. The patient was not very compliant with her treatment and received her first appropriate ICD discharge due to ventricular fibrillation (266 beats per minute) at the age of 24 years. Therefore, another catheter ablation was performed with a temporary improvement in symptoms. After two years, her symptoms returned, and additional electrophysiological testing revealed polymorphic PVCs originating from the right ventricle. This time, catheter ablation was ineffective. Amiodarone was replaced with 240 mg of sotalol per day. Holter 24 h monitoring was performed without medical therapy discontinuation, and 31,000 (30%) polymorphic PVCs, episodes of bigeminy and trigeminy, 2000 ventricular couplets, 200 triplets, and 44 episodes of NSVT episodes were registered (Figure 2). She was prescribed 200 mg of amiodarone and 100 mg of metoprolol per day. Her status improved: she was asymptomatic again and her repeated 24 h Holter monitoring registered 1072 (1%) PVCs. As higher arterial blood pressure was reported in the evenings, 30 mg of zofenopril was initiated.

**Figure 3:**
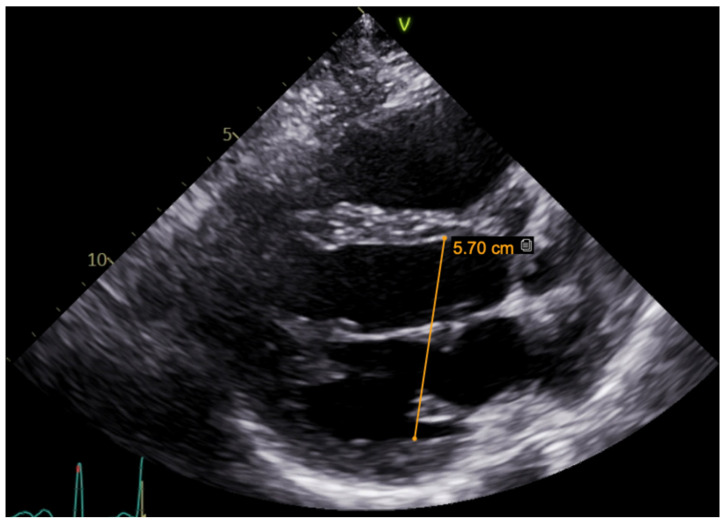
TTE parasternal long-axis view before treatment (moderately dilated left ventricle, LVdd 57 mm).

**Figure 4:**
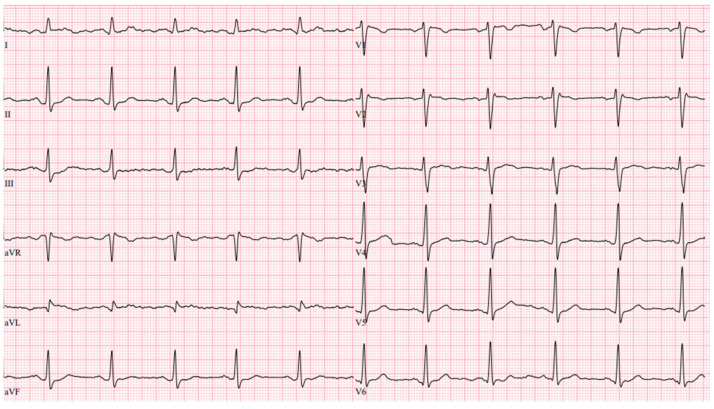
Twelve-lead ECG during combination treatment (no PVCs).

**Figure 5:**
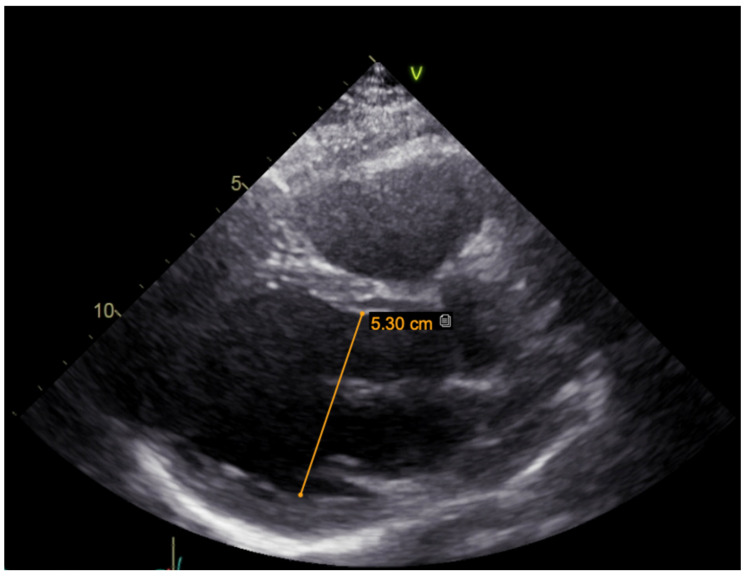
TTE parasternal long-axis view after combination treatment (improvement in LV function, LVdd 53 mm).

A hereditary disease was suspected, as the patient’s father died suddenly at the age of 40. Thus, targeted exonic sequencing of the *SCN5A* gene was performed. The pathogenic variant of the SCN5A gene (NM_198056.2) *c.2440C* > T (p.(Arg814Trp); rs199473161) was identified in a heterozygous state. The variant was advised to be evaluated according to clinical phenotype. It was decided to specify structural changes with repeated cardiac magnetic resonance imaging, although this scan was uninformative due to artefacts of ICD leads. Thus, TTE was repeated. No significant LV dilation (LVdd 54 mm) or systolic dysfunction (LV EF Simpson Biblane > 55%) were visible. The ergometry bicycle test demonstrated PVCs provoked by exercise, with most of them being monomorphic. A computerized tomography (CT) coronary angiogram ruled out coronary artery pathology, as the measured Agatston score was zero. During this time, the patient was on amiodarone and beta blockers. At the age of 31, the patient developed thyrotoxicosis and amiodarone had to be discontinued. Due to aggravated PVC symptoms, metoprolol was changed to 10 mg of bisoprolol per day. However, despite an optimized dose of beta blockers, the burden of PVCs increased, and approximately 66,491 (44%) PVCs were registered during 24 h Holter monitoring. TTE revealed a moderately dilated left ventricle (LVdd 57 mm, Figure 3) and impaired systolic function (LV EF 44%) in the context of ventricular bigeminy. A shared consultation with experts from the European Reference network of Rare Diseases was arranged, and a diagnosis of MEPPC syndrome was determined. Flecainide at a dose of 100 mg BID was added to bisoprolol at a dose of 10 mg. After several days, the dose of flecainide was increased to 300 mg a day, but no significant improvement in the patient’s status was observed. Thus, mexiletine was added, with the dose gradually titrated up to 150 mg three times a day. A substantial effect was seen in telemetry and registered in the ECG (Figure 4). All mentioned treatment adjustments were made during hospitalization in the arrhythmias department of Santaros Clinics, under telemetry surveillance, and with continuous monitoring of the patient’s clinical status. The patient was discharged with medical therapy of 7.5 mg of bisoprolol, 300 mg of flecainide, and 450 mg of mexiletine per day. During a follow-up visit after 1 month, a significant reduction in symptoms (fewer palpitations and better physical exercise tolerance), an improvement in LV function (LV EF by Simpson Biplane in repeated TTE was 53% and LVdd was 53 mm, Figure 5), and only 5819 (5%) PVCs under 24 h monitoring were observed. After 3 months, a further reduction to 858 (1%) PVCs under 24 h monitoring was registered and the patient had no complaints of palpitations. Dosages of antiarrhythmic drugs were reduced and as remission remained, discontinuation of mexiletine is under consideration.

## 4. Discussion

The first description of MEPPC syndrome by Laurent and colleagues in 2012 involved 21 individuals from three unrelated families [1]. To date, only 11 articles have been published on the topic, including three case reports, leaving significant knowledge gaps [6,8].

MEPPC syndrome typically presents with palpitations, dyspnea, and syncope, and can sometimes lead to life-threatening arrhythmias such as ventricular tachycardia or sudden cardiac death [2,3,4,5,6,7]. Often, premature atrial complexes, atrial fibrillation, and varying conduction abnormalities, together with accelerated junctional rhythms, are concomitant [1,2]. In untreated cases, symptoms of heart failure due to dilation of the left ventricle or impaired systolic function are relevant [8]. In our case, the syndrome manifested with palpitations, fatigue, and intolerance to physical activity. The progression of symptoms correlated with a high burden of PVCs and decreased LV EF.

The origin of DCM remains a subject of debate in the literature. Some authors attribute coexisting cardiomyopathy to a high burden of ectopic beats, suggesting its reversibility with medical therapy [1,2,3,4,5,6,7,8,9]. Conversely, others emphasize the impact of specific genetic mutations on cardiac structural proteins, sarcomere proteins, nuclear membrane proteins, and ionic homeostasis imbalance [4]. Models of Purkinje fiber and ventricular cell dynamics suggest that the SCN5A gene variant induces rate-dependent ectopic activity from Purkinje fibers, potentially contributing to dilated cardiomyopathy. Carriers of this mutation showed modest responses to standard heart failure treatments. However, PVCs and DCM were significantly reduced with sodium channel blockers, such as amiodarone or flecainide [9]. On the other hand, MEPPC syndrome is caused by gain-of-function mutations in the SCN5A gene, which encodes the NaV1.5 sodium channel, crucial for generating the inward sodium current (INa) in excitable cells. This INa drives rapid depolarization, which is essential for action potential initiation and propagation [10]. The mutations are concentrated in the voltage-sensitive domain of NaV1.5, forming gating pores that allow the passage of cations, especially sodium and potassium. This leads to acidosis, calcium overload, and reduced sarcomere sensitivity to calcium. Persistent sodium currents may also contribute, explaining the pathology of DCM-linked NaV1.5 mutations [4]. Although clinical genetic testing for cardiomyopathy has been available for over a decade, the number of genes identified as disease-causing has surged significantly in recent years, frequently lacking strong supporting evidence [11]. A definitive genetic diagnosis can be determined in 29% of patients with arrhythmogenic disorders. Most pathogenic variants (97%) were found in the 13 well-established core genes: KCNQ1, KCNH2, SCN5A, RYR2, MYBPC3, MYH7, TNNT2, TNNI2, LMNA, TTN, PKP2, DSP, and DSG2 [12]. The association between arrhythmia and cardiomyopathy with voltage sensor mutations was first reported in 2005 by Olson et al. They screened patients with idiopathic dilated cardiomyopathy and identified five SCN5A mutations, including a novel voltage sensor mutation (R814W). This finding highlighted the critical role of voltage sensor mutations in cardiac diseases [13]. Two years later, a family case study was published describing a novel SCN5A missense mutation, R814Q, in the S4 transmembrane segment, presenting in the homozygous state and resulting in sustained monomorphic ventricular tachycardia [14]. The R814W mutation was later confirmed to be linked to DCM arrhythmia syndrome through a study where the mutation was engineered into a human heart sodium channel and coexpressed in kidney cells with the β1 subunit [15]. Another article described a mutation phenotype characterized by nonischemic dilated cardiomyopathy (DCM) together with non-sustained ventricular tachycardia [16]. A study confirmed that replacing arginine with tryptophan significantly disrupts the S4 segment’s structure and movement, causing it to twist due to tryptophan’s strong interaction with lipid tails [17]. In our case, the pathogenic variant of the SCN5A gene, leading to arginine-to-tryptophan substitution in position 814, has been identified in a heterozygous state.

The diagnosis of the disease requires a comprehensive cardiovascular examination and genetic testing. The pivotal finding is polymorphic PVCs in ECGs and 24 h Holter monitoring or bicycle ergometer ECGs [3]. This syndrome does not affect beats originating from the normal sinus node, and as a result, ST segment morphology and the QT interval remain normal. Transthoracic echocardiography is instrumental in evaluating left ventricular function. Regular TTE monitoring is essential for early detection of arrhythmia-related cardiomyopathy, particularly when primary sonographic measurements are normal. Cardiac magnetic resonance may reveal ventricular fibrosis in certain cases [2].

Clinical presentation and diagnostic approaches lack specificity, underscoring the absence of defined diagnostic criteria for MEPPC. Patients with a positive family history of arrhythmias or sudden cardiac death warrant special attention. Genetic testing should be considered in all indeterminate cases [11]. In our case, a pathogenic SCN5A gene variant was identified, which is crucial for diagnosis and treatment selection.

According to current guidelines, catheter ablation is recommended as the first-line treatment for symptomatic idiopathic PVCs originating from the right ventricular outflow tract (RVOT) or the left fascicles [18]. However, in MEPPC syndrome, ablation therapy exhibits limited efficacy due to the multifocal nature of PVCs emanating from the entire Purkinje system and medical therapy has substantial superiority [6]. The literature predominantly describes the efficacy of class I AADs, specifically flecainide and quinidine [2]. In our case, most of the PVCs originated from the RVOT or right ventricle and after each catheter ablation, a new foci started to prevail, leading to multiple procedures. Only a triple therapy of beta blockers and a combination of two class I AADs has shown a significant reduction in arrhythmia burden. In the literature, mexiletine monotherapy is ineffective, but its addition to quinidine leads to a reduction in PVC burden to less than 10 percent [19]. Quinidine is an orphan drug; thus, we tried combinations of other class I AADs. There is a reported case of an SCN5A mutation in a child with amiodarone-responsive multifocal ventricular ectopy-associated cardiomyopathy [20]. We also saw an improvement in symptoms under treatment with beta blockers and amiodarone, but the drug’s toxicity makes long-time treatment with amiodarone questionable [12,14]. There are data suggesting beneficial concomitant overdrive pacing for controlling severe arrhythmia [19]. The triggered ventricular action potentials disappeared at higher pacing frequencies (2 Hz) [1].

As MEPPC syndrome is a very rare and recently recognized entity, there is a lack of high-value studies available. Consequently, the precise prognosis of patients with this condition remains unknown. A family history of ventricular arrhythmias often indicates a poor prognosis and an increased risk for SCD [8]. In our opinion, this presupposes that the indications for ICD in MPPC patients should be carefully evaluated and adjusted for the individualized risks of specific cases.

## 5. Conclusions

MEPPC syndrome, characterized by a gain-of-function mutation in SCN5A, presents unique clinical patterns and treatment challenges due to its high burden of arrhythmia and resistance to standard drug treatment or catheter ablation. Our case study illustrates the diagnostic and therapeutic journey of a patient with MEPPC syndrome, highlighting the importance of genetic testing and tailored therapy as delayed treatment may lead to LV dysfunction. Special attention should be given if a hereditary disease is suspected, as in this era of genetic testing, prompt detection of mutations can help avoid ineffective treatments. As indicated by most of the literature, the treatment approach in MEPPC typically involves a combination of class I antiarrhythmic drugs. Our case represents the importance of multidisciplinary collaboration in managing such rare syndromes, as a successful outcome was achieved after consulting with specialists from Rare Diseases and European Reference networks and the consensus to initiate treatment with a combination of class I antiarrhythmic drugs with beta blockers was made. This case raises the question of whether adding beta blockers might be beneficial in instances of drug-resistant MEPPC. Consequently, further research involving larger cohorts and long-term follow-up is necessary to ascertain the efficacy and safety of this adjunctive therapy.

## Data Availability

No new data were created or analyzed in this study. Data sharing is not applicable to this article.
